# Diagnosis support systems for rare diseases: a scoping review

**DOI:** 10.1186/s13023-020-01374-z

**Published:** 2020-04-16

**Authors:** Carole Faviez, Xiaoyi Chen, Nicolas Garcelon, Antoine Neuraz, Bertrand Knebelmann, Rémi Salomon, Stanislas Lyonnet, Sophie Saunier, Anita Burgun

**Affiliations:** 1 Centre de Recherche des Cordeliers, INSERM, Université de Paris, Sorbonne Université, F-75006 Paris, France; 2 Institut Imagine, Université de Paris, F-75015 Paris, France; 3 Département d’informatique médicale, Hôpital Necker-Enfants Malades, Assistance Publique - Hôpitaux de Paris (AP-HP), F-75015 Paris, France; 4 Service de Néphrologie Transplantation Adultes, Hôpital Necker-Enfants Malades, F-75015 Paris, France; 5 Université de Paris, F-75006 Paris, France; 6 Institut Necker-Enfants Malades, INSERM, Hôpital Necker-Enfants Malades, F-75015 Paris, France; 7 Service de Néphrologie Pédiatrique, Hôpital Necker-Enfants Malades, Assistance Publique-Hôpitaux de Paris (AP-HP), Université de Paris, F-75015 Paris, France; 8 Laboratory of Embryology and Genetics of Congenital Malformations, INSERM UMR 1163, Université de Paris, Imagine Institute, F-75015 Paris, France; 9 Service de génétique, Hôpital Necker-Enfants Malades, Assistance Publique - Hôpitaux de Paris (AP-HP), F-75015 Paris, France; 10 Laboratory of Renal Hereditary Diseases, INSERM UMR 1163, Université de Paris, Imagine Institute, F-75015 Paris, France; 11 PaRis Artificial Intelligence Research InstitutE (PRAIRIE), Paris, France

**Keywords:** Scoping review, Rare disease, Genetic diseases, Diagnosis, Clinical decision support, Artificial intelligence, Machine learning, Patient similarity, Phenotype

## Abstract

**Introduction:**

Rare diseases affect approximately 350 million people worldwide. Delayed diagnosis is frequent due to lack of knowledge of most clinicians and a small number of expert centers. Consequently, computerized diagnosis support systems have been developed to address these issues, with many relying on rare disease expertise and taking advantage of the increasing volume of generated and accessible health-related data. Our objective is to perform a review of all initiatives aiming to support the diagnosis of rare diseases.

**Methods:**

A scoping review was conducted based on methods proposed by Arksey and O’Malley. A charting form for relevant study analysis was developed and used to categorize data.

**Results:**

Sixty-eight studies were retained at the end of the charting process. Diagnosis targets varied from 1 rare disease to all rare diseases. Material used for diagnosis support consisted mostly of phenotype concepts, images or fluids. Fifty-seven percent of the studies used expert knowledge. Two-thirds of the studies relied on machine learning algorithms, and one-third used simple similarities. Manual algorithms were encountered as well. Most of the studies presented satisfying performance of evaluation by comparison with references or with external validation. Fourteen studies provided online tools, most of which aimed to support the diagnosis of all rare diseases by considering queries based on phenotype concepts.

**Conclusion:**

Numerous solutions relying on different materials and use of various methodologies are emerging with satisfying preliminary results. However, the variability of approaches and evaluation processes complicates the comparison of results. Efforts should be made to adequately validate these tools and guarantee reproducibility and explicability.

## Introduction

There are more than 7000 rare diseases affecting approximately 350 million people worldwide. Eighty percent of rare diseases are genetic diseases. According to a report from Globalgenes [1], most clinicians have limited knowledge about these diseases, and 40% of general practitioners and 24% of specialist doctors do not have time to work on these diagnoses. All these factors lead to underdiagnosis or delayed diagnosis of rare diseases. Moreover, even if the patient is suspected of suffering from a rare disease, there is still a large possibility of misdiagnosis because of the overlapping spectrum of symptoms of many rare diseases [2]. In general, final diagnosis for most rare diseases is performed using a genetic test that tends to be focused on a small set of diseases. Given all these constraints, a recent review concluded that rare disease diagnosis is still a challenging task [3].

Considering the growing complexity of medical knowledge and the increasing availability of data sources such as electronic health reports (EHRs), many decision support systems have been developed to assist clinicians in their decision-making, particularly for diagnosis and prediction tasks [4]. The objectives of these diagnosis support systems can be different: more widespread, more accurate, more effective, less expensive or less time consuming. Some tools are focused on a specific rare disease or a specific group of rare diseases, while other tools aim to provide general diagnosis support for all rare diseases. According to different objectives, various data sources and methods were considered, and the evaluation processes were even more diverse.

In 2019, Montani and Striani [5] reviewed clinical decision support tools using artificial intelligence (AI). They considered two categories of AI: knowledge-based AI, using a “top-down” fashion based on human knowledge, and data-driven AI, using a “bottom-up” fashion to generate knowledge from a large amount of data. Knowledge-based AI aims to model expert knowledge with artifacts such as ontologies and rules and operationalize it in terms of software or algorithms for reasoning and solving problems, whereas in the case of data-driven systems, models allowing classification and prediction are derived from the processing of data provided to the system. Montani and Striani [5] identified 13 studies proposing decision support dealing with diagnosis. Among these, 11 were exclusively knowledge-based systems, and 2 adopted knowledge-based methods in combination with data-driven methods. However, this review was not specified to rare disease diagnoses.

In this review, we aim to (i) present all the initiatives seeking to support the diagnosis of rare diseases considering the plurality of objectives (making diagnosis more accurate, more widespread, less expensive, etc.), the multiplicity of materials (clinical signs and symptoms, fluids, medical images, etc.), and all methodological approaches (e.g., automatic algorithms or methods based on manually generated scores or decision trees), and (ii) provide an intensive discussion of the characteristics of ready-to-use systems.

## Methods

This scoping review was performed following the recommendations from Arksey and O’Malley [6].

### Identifying the research question

In this scoping review, we considered rare diseases as diseases described as rare within the studies under review, diseases with prevalence less than 1/2000, or diseases present in the Orphanet list [7]. Both postnatal and antenatal situations were considered. Our objective was to identify and analyze articles that report on using algorithms or computer-aided systems to support the diagnosis of rare diseases. We included in this review all publications using fully automated approaches and publications using more traditional or empirical knowledge modeling (manually generated scores, decision trees, etc.), which qualified as “manual” in the rest of this article.

### Identifying relevant studies

Two categories of articles were considered: (i) articles published in medical and health-related journals and (ii) articles published in computer science and AI journals/conferences with applications in the diagnosis of rare diseases. We limited ourselves to a 10-year period, from January 1, 2009 to August 31, 2019, considering that older articles may not be relevant for our analysis. Only studies written in English and related to humans were considered. Reviews were excluded.

The search strategy was defined to identify relevant studies from these two categories. PubMed was used to search the MEDLINE database, covering biomedicine and health care, as well as bioinformatics and some AI journals indexed in MEDLINE, such as Artificial Intelligence in Medicine. This search was then complemented by the exploration of Web of Science (WoS) to identify methodological publications that were not indexed in MEDLINE, such as Journal of Artificial Intelligence Research (JAIR) and Artificial Intelligence Journal (AIJ). Three additional major AI conference websites were further explored independently, as they were not included in MEDLINE or Web of Science: Neural Information Processing Systems (NeurIPS), Association for the Advancement of Artificial Intelligence (AAAI) and International Joint Conferences on Artificial Intelligence (IJCAI).

#### Identifying medical and health-related publications

Our search strategy was defined as three co-occurring concepts: “diagnosis”, “rare diseases” and “support tool” (see Table 1). A set of synonymous terms was identified for each notion using an iterative process. For the diagnosis concept, we included terms such as “diagnoses” or “diagnostic”. For the rare diseases concept, we also considered “orphan” diseases and “genetic” diseases. The search strategy for the support tool notion was complex due to the heterogeneity of methods and vocabulary that were used by authors. The selected terms had to be broad enough to identify the maximum number of relevant publications and specific enough to reduce the number of false positives. Selected terms included AI vocabulary such as “artificial intelligence”, “decision support”, “expert system”, and “information retrieval”. For PubMed, Medical Subject Heading (MeSH) terms were included in the query along with keywords from titles and abstracts.

**Table 1:** Database queries

Database	Query
PubMed	**(((**“Diagnosis”[MeSH] OR “diagnostic”[TIAB] OR “diagnostics”[TIAB] OR “diagnosis”[TIAB] OR “diagnoses”[TIAB]**) AND** **(**“rare diseases”[MeSH] OR “Genetic Diseases, Inborn/genetics”[MeSH] OR ((“rare”[TIAB] OR “genetic”[TIAB] OR “orphan”[TIAB]) AND (“diseases”[TIAB] OR “disease”[TIAB]))**)) OR** **(**“Rare Diseases/diagnosis”[MeSH] OR “Genetic Diseases, Inborn/diagnosis”[MeSH]**)) AND** **(**“Decision Support Systems, Clinical”[MeSH] OR “Decision Support Techniques”[MeSH] OR “decision support”[TIAB] OR “artificial intelligence”[MeSH] OR “artificial intelligence”[TIAB] OR “Medical Informatics Computing”[MeSH] OR “Big data”[MeSH] OR “Data Mining/methods”[MeSH] OR “expert system”[TIAB] OR “information retrieval”[TIAB] OR “search engine”[MeSH] OR “Software Design”[MeSH] OR “Software Validation”[MeSH]**)**
Web of Science	**(**ALL = **(**“rare disease*” OR “genetic disease*” OR “orphan disease*”**) OR (**TI = **(**“rare” OR “genetic” OR “orphan”**) AND** TI = “disease*”**)) AND** ALL = **(**diagnosis OR diagnostic* OR diagnoses**) AND** ALL = **(**“decision support*” OR “expert system*” OR “artificial intelligence” OR “information retrieval” OR “search engine*” OR “medical informatics computing” OR “software design” OR “software validation” OR “big data” OR “data mining”**)**

Additionally, we used a snowball strategy to find other relevant publications: we completed our search by screening bibliographies from relevant studies and looked for “similar articles” suggested by PubMed.

#### Identifying methodological publications

As Web of Science does not provide MeSH term matching, the full search strategy was adapted using keywords for identifying methodological publications in the domain of computer science and AI (Table 1). For the three additional major AI conference websites, searches were conducted on the websites or using Google search restricted to the considered websites. Selected articles had to address the notions of “rare disease” and “diagnosis”. Similar to PubMed, we completed our search by screening bibliographies from relevant studies.

### Study selection

One reviewer (CF) screened all the titles and abstracts and scored them from 0 (exclude) to 2 (keep). Publications that scored 1 (not sure) were then collectively reviewed by CF, XC and AB until a consensus was reached, and then all articles were classified as 0 or 2.

Exclusion criteria were discussed among the three reviewers and definitively set once 10% of the abstracts were screened. We excluded publications:

- Aimed at assessing disease severity, survival, prognosis or risk for recurrence but not diagnosis (publications identifying primary risk for a disease were kept)
- Aimed at assessing the risk for a disease using only environmental factors
- Aimed at identifying the best treatment option based on individual variability
- Aimed at classifying diseases without performing a more precise diagnosis/subtyping (e.g., for cystic fibrosis, assessing the thickness of airways)
- Aimed at improving disease knowledge (e.g., aiming at identifying gene signatures) instead of generating a diagnosis tool or algorithm
- Focusing on diseases that are neither rare nor genetic (e.g., Alzheimer, Parkinson)

All publications that scored 2 at the end of the selection process were read in their entirety, and information of interest was extracted and collected using a specific form.

### Charting the relevant studies

A standardized charting form was established to synthetize relevant publications. The information of interest can be categorized in four main sections: metadata, publication scope, algorithm and model, implementation of the diagnosis support tool.

The “Metadata” section consisted of publication title, date, authors, country and source (PubMed or WoS or conference website). The “Publication scope” section aimed to summarize information such as the main objective and targeted diseases of the article and data type (e.g., image, phenotype concepts) and volume. In this section it was also specified if the system was developed for ante or postnatal diagnosis. If relevant, the data encoding was specified. In the “Algorithm and model” section, we described the methods, including the kind of knowledge that was involved in the model (prior knowledge or not), and the evaluation process, including metrics of performances. The “Implementation” section focused on the technical characteristics and functionalities of the system. An intensive discussion on ready-to-use tools, advanced algorithms and prototypes of interest is provided.

One reviewer (CF) charted all the selected publications. The three reviewers (CF, XC, AB) met to resolve uncertainties.

### Collating, summarizing and reporting the results

The results from the data charting were summarized and analyzed to present an overview of the methods and results encountered.

## Results

We retrieved 829 articles from PubMed, 89 articles from Web of Science (excluding duplicates retrieved from PubMed) and 55 articles from AI conference websites, for a total of 973 articles. Screening of titles and abstracts of these articles was conducted to identify 51 relevant articles from PubMed, 11 additional publications from Web of Science and one from the IJCAI website. Of note, 19% of the relevant publications (12 out of 63) were not indexed in MEDLINE.

**Fig. 1:**
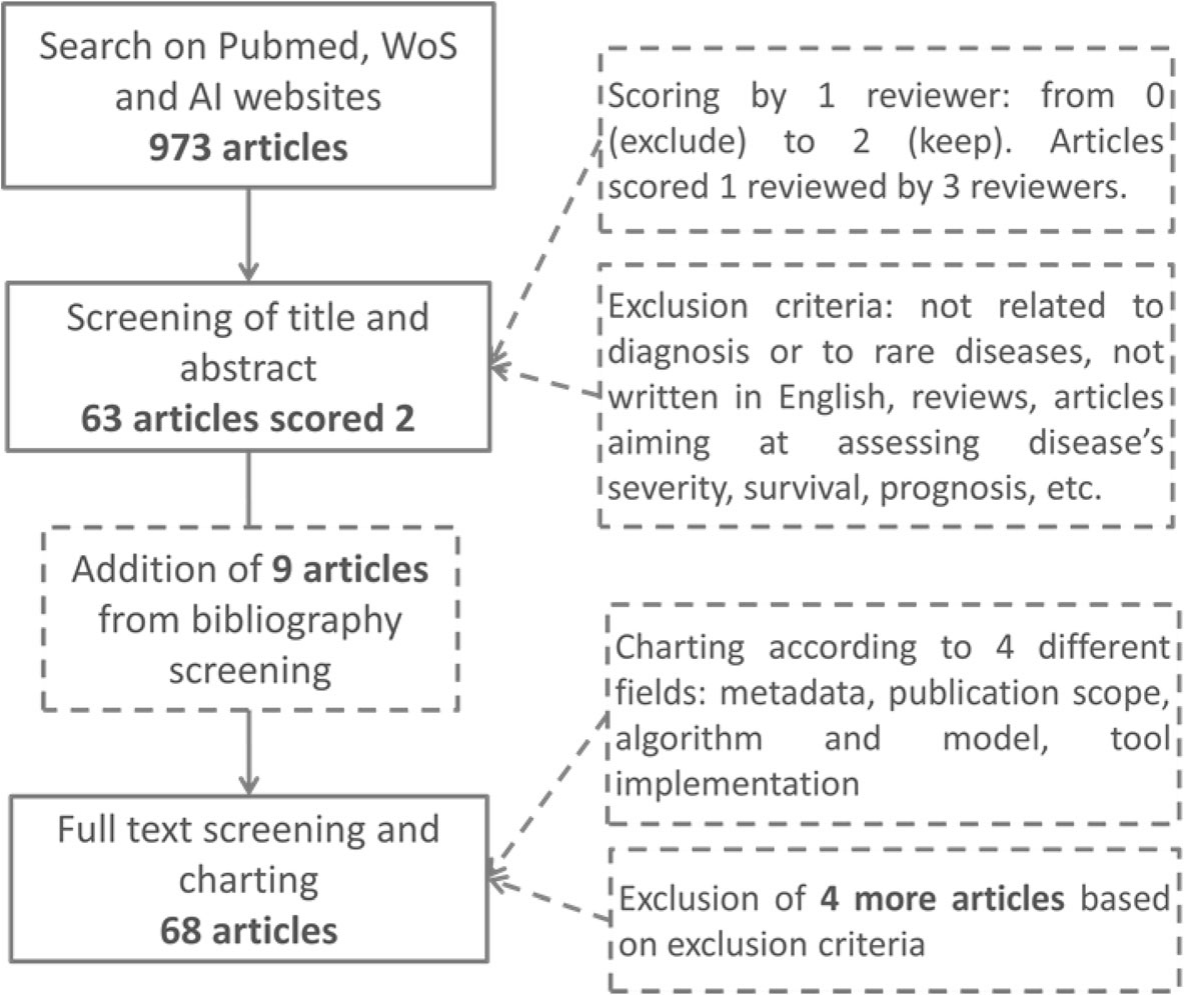
Flowchart of the screening process

We identified 9 more articles through the snowball strategy. These 72 articles were fully read and charted. Four articles were excluded at the end of this process with an agreement of the three reviewers. More precisely, we excluded studies aiming to assess severity [8], to assess longitudinal data of cases and controls [9], describing big data management software with one example on genetic disease [10] and aiming to identify “noncommon” diseases without performing a diagnosis [11]. Sixty-eight articles were consequently retained at the end of the whole process (Fig. 1).

### Metadata

Analysis of metadata shows that rare disease diagnosis support has become an active research topic. Among the 68 articles published between 2009 and 2019, more than 50% were published since 2016. Regarding authors’ affiliations, Europe and North America were the major contributors: 37 articles (54%) had at least one coauthor from Europe, and 21 (31%) had at least one author from North America. The most represented country was Germany (16/68 studies). Sixty-one articles were published in life science and bioinformatics journals, and 7 were published in methodological journals (e.g., IJCAI, Electronic Physician journal).

### Publication scope

This section aimed to provide insights into the article by identifying the target (targeted patients and disease) and the considered material (data nature and volume). These results are described in the two following subsections.

#### Publication target

Regarding the targeted patient, two different contexts were focused on, i.e., postnatal or antenatal diagnosis. Most of the articles (61/68) focused on diagnosis after birth, while 7 studies consisted of prenatal screening for fetal syndromes [12], diseases with chromosomal abnormalities [13], aneuploidies [14–16] and trisomy [17, 18] based on noninvasive markers (demographics, sonographic markers, maternal blood). The two contexts are referred to in the following sections as “the post-natal studies” and “the prenatal studies”. We focused on 61 postnatal studies.

Regarding the diseases under study, an important variability in the number of targeted diseases was identified. Articles were categorized into three groups based on the number of targeted diseases:

- **Group 1 - Studies focusing on one disease** (e.g., Huntington disease): 29 studies out of 61 (48%) [19–47];
- **Group 2 - Studies focusing on a class of diseases or syndromes** (e.g., macular diseases, facial genetic dysmorphologies): 15 studies (25%) [48–62];
- **Group 3 - Studies focusing on the whole spectrum of genetic/rare diseases**: 17 studies (28%) [2, 63–78].

The most individually studied diseases were thalassemia [20, 28, 29, 47], Down syndrome [21, 30, 34], cystic fibrosis [46, 48, 52], Marfan syndrome [25, 27] and Huntington disease [19, 33].

#### Material

Material nature and data volume were assessed for each study.

##### Material nature

Diagnosis support was performed on various types of material (Table 3). Sixteen studies (26%) used images, 12 studies (20%) used quantitative data from laboratory test results on fluids (blood, plasma or urine), and 22 studies (36%) used other types of phenotypes, namely, concepts extracted automatically from narrative reports in EHRs or case reports. In the rest of this article, we use the term “phenotype concepts” to refer to the last category. Other types of material were also considered, such as ad hoc questionnaires (3 studies) [52, 53, 58] and combinations of clinical features and family history (8 studies).

**Fig. 2:**
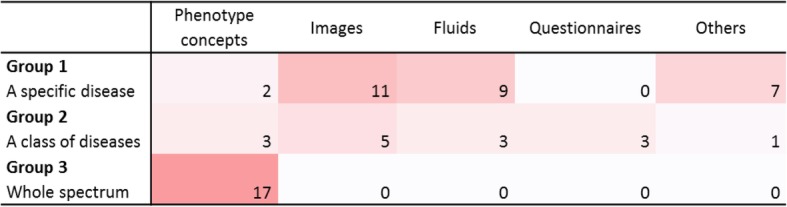
Correlations between the number of targeted diseases and material nature. All studies directed to all rare/genetic diseases were based on phenotype concepts. Studies directed to a class or one specific disease could take advantage of disease-related materials such as images or fluids

Some correlations were identified between the nature of the material and the number of targeted diseases (Fig. 2). Not surprisingly, publications targeting the whole spectrum of rare diseases were all based on phenotype concepts (17 articles).

##### Data volume

Regarding data volume, the number of patients included in each study was assessed. The data volume information was present in 80% of the publications (49/61). The results are summarized in Table 2. All publications targeted to one specific disease (group 1) used control datasets in addition to the patient sets. Regarding the publications targeted to one class of diseases (group 2), several datasets could be encountered within one study. In that case, the data volume was assessed for each dataset independently. For the publications targeted to all rare diseases (group 3), simulated patients were sometimes used for evaluation. We excluded the simulations in the analysis of data volume and considered only the number of real patients. For studies relying on images or fluids, if the number of patients was not clearly specified, we considered the number of images or samples.

**Table 2:** Number of patients and controls per dataset

	Number of studies	Number of studies with datasets	Number of datasets	Number of patients	
				Median	Mean [Min, Max]
**Patients**					
Group 1	29 studies	27 studies	29 datasets	50	291 [7, 5050]
Group 2	15 studies	14 studies	20 datasets	98	730 [5, 10,593]
Group 3	17 studies	8 studies	10 datasets	161	6929 [40, 39,000]
**Controls**					
Group 1	29 studies	27 studies	29 datasets	70	105,491 [10, 2,966,363]

Studies are grouped according to the number of diseases they address. Group 1: one disease; group 2: a class of diseases; group 3: all rare/genetic diseases. The number of studies, datasets and patients per dataset for each group is given. For group 1, the number of individuals in the control groups is also given. Datasets from studies addressing all rare diseases (group 3) contain more patients on average

The number of patients could vary from less than 20 patients [20, 21, 24, 35, 40, 43, 55] to more than 1000 patients [37, 47, 51, 62, 72, 75]. As expected, the number of patients was usually more important for publications targeting more than one disease (groups 2 and 3) than for publications targeting one specific rare disease (group 1) (Table 2). Only two studies from group 1 considered more than 1000 patients, familial hypercholesterolemia [37] and beta thalassemia [47].

### Algorithm and model

This section aims to describe how diagnosis support was performed with different algorithms and models. We focused on (i) preprocessing, (ii) developed methods, and (iii) evaluation and validation.

#### Preprocessing

Many studies have considered preprocessing steps to improve the performance of the algorithms. The most frequent was feature extraction. Thirty-three out of 61 studies (54%) described in their methodology a feature extraction process based on dimension reduction, selection of a subpart of relevant features, or comparison of the selection of features from different natures or databases (e.g., phenotype concepts and genotypes [73], addition of demographics [56], clinical and biochemical phenotype concepts [54], clinical notes or MEDLINE [75]).

#### Developed models

We distinguished between knowledge-based approaches (including prior knowledge from experts, literature, ontologies) and data-driven approaches [5] to categorize the diagnosis-supporting models. Knowledge-based approaches ranged from simple decision trees created by experts based on their knowledge of a disease to more sophisticated models using disease and phenotype ontologies to support diagnosis. Data-driven approaches included all models directly derived from data, such as algorithms using images or fluids trained to classify patients based on features extracted from raw data. In the case of the addition of any prior knowledge to the data-driven approach, the system was qualified as a hybrid.

**Fig. 3:**
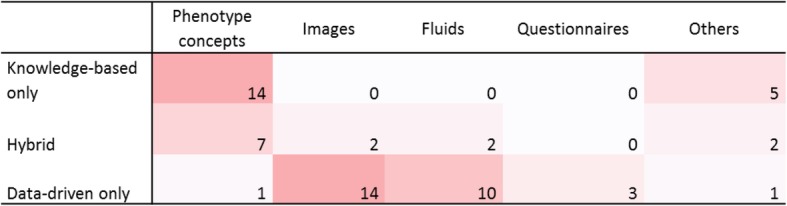
Correlations between the knowledge model and material nature. Knowledge-based models were based on phenotype concepts or combinations of clinical features. Data-driven models were mostly based on images or fluids

Based on this categorization, three-fourths of the systems among the 61 postnatal studies were based on a single approach: 19 studies (31%) used knowledge-based models exclusively, and 29 studies (48%) used data-driven models. Hybrid models were encountered in 13 articles, corresponding to 21% of the publications (Fig. 3 and Table 3).

**Table 3:** Publication summary

Material	Knowledge	Machine learning	Articles
Phenotype concepts (22 studies)	Knowledge-based (14 studies)	No	[2, 54, 55, 61, 63–71, 77]
	Hybrid (7 studies)	Yes	[27, 73–76, 78]
		No	[25]
	Data driven (1 study)	Yes	[72]
Fluids (12 studies)	Hybrid (2 studies)	Yes	[28, 29]
	Data driven (10 studies)	Yes	[20, 23, 24, 31, 36, 42, 47, 48, 56, 60]
Images (16 studies)	Hybrid (2 studies)	Yes	[41, 45]
	Data driven (14 studies)	Yes	[19, 21, 22, 30, 33–35, 43, 44, 49, 51, 57, 59, 62]
Questionnaires (3 studies)	Data driven (3 studies)	Yes	[52, 53, 58]
Family history and combined material (8 studies)	Knowledge-based (5 studies)	No	[26, 32, 38, 46, 50]
	Hybrid (2 studies)	Yes	[37, 39]
	Data driven (1 study)	Yes	[40]

References are listed in column “articles” according to the type of material considered and the model used (presence/absence of prior knowledge and of machine learning). The number of studies according to material and knowledge is given in parentheses

**Fig. 4:**
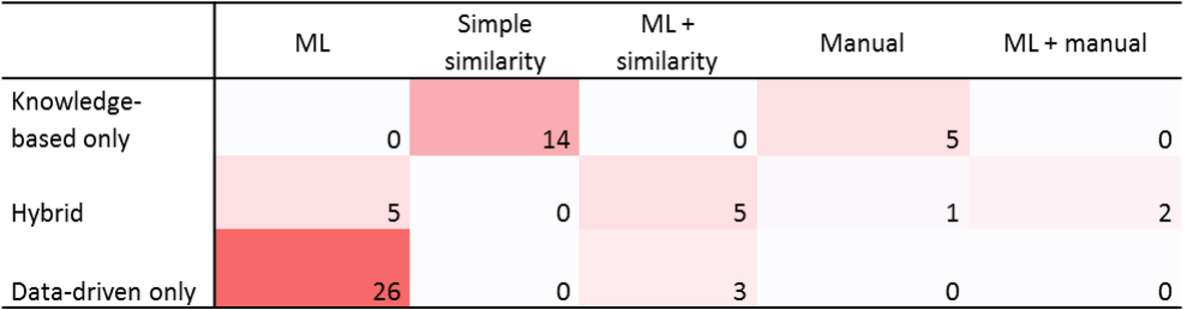
Correlations between the knowledge model and the methods. Data-driven systems were all based on machine learning (associated or not to simple similarity measurement). Knowledge-based systems were either based on simple similarity or manually generated algorithms

Among the 19 studies based exclusively on prior knowledge, 5 systems (26%) were based on manually acquired knowledge and simple representation, such as manually designed decision trees [26, 38, 50], rather than automated approaches. The remaining 14 studies (74%) consisted of modeling the disease by the presence or frequencies of phenotype concepts and then applying a simple similarity method (Fig. 3 and Fig. 4). The knowledge source could be expert knowledge, literature or existing knowledge bases, such as Orphanet [79] or Online Mendelian Inheritance in Man (OMIM) [80]. As expected, 12 studies re-used the Human Phenotype Ontology (HPO) [81] as a knowledge source for phenotype coding, and 8 also used the tree structure of HPO to address granularity issues and calculate semantic similarity metrics. For these 14 studies, decision support was as follows: each disease is described by a set of phenotype concepts that correspond to the signs and symptoms of the disease. Possible diagnoses of a new patient are then scored by comparing the phenotypic description of the patient to such knowledge using similarity metrics such as cosine. The diagnosis support system then returns a list of diseases ranked by the similarity score for each patient. Three studies [65, 67, 68] out of 14 included gene-disease knowledge in their model. One of these systems [67] needed as input the list of the patient’s phenotype concepts complemented by the list of variants identified in the patient’s genome.

Among the 29 systems based exclusively on data, 86% used images (14 studies) or fluids (10 studies) (Fig. 3). All 29 studies used “machine learning” (ML), which can be considered the ability to learn without explicitly being programmed. In this review, we considered a broad acceptance of ML, from simple statistical methods such as regression to deep learning, if a training phase is considered. As for deep learning, we also considered a broad acceptance of this term and we included all systems mentioned using neural networks. Support vector machine (SVM) was the most popular method, corresponding to 10 studies. Deep learning was used in 6 studies using images [44, 57, 59, 62] or fluids [20, 31] and exhibited good performance on such data. Associations of different algorithms, such as fusion algorithms, were encountered in 2 studies [52, 53]. Other authors reported using more traditional statistical modeling, such as regression [24] or decision trees [42, 60]. The K Nearest Neighbors algorithm was used in 2 studies [51, 72]. Three studies [43, 58, 72] also used simple similarity methods before applying machine learning models (Fig. 4). Two publications were based on transfer learning, with models built and trained on a domain and then transferred to a new domain [82]. The first study used MRI images from healthy people to train an algorithm for autism spectrum detection [57]. The second study aimed to identify genetic syndromes on photographs based on training for face recognition [62].

For the 13 studies using hybrid systems, the most frequent combination (8 studies) was initial knowledge-based feature selection followed by data-driven models. The latter part could be either machine learning models [28, 29, 37, 41, 45] or data-driven processes for feature weighting or dimension reduction to create scores [25, 27, 39]. Two studies [73, 78] used a combination of similarity calculation between the patient’s phenotype concepts and the knowledge-based description of a disease on the one hand and machine learning on the other hand. Another type of combination consisted of combining similarity between patients and text-mined literature on the one hand and similarity metrics among patients on the other hand [75]. In these frameworks, deep learning was used in 4 studies [28, 29, 45, 74] and SVM in one study [41]. One study [73] used a fusion algorithm. In the same way as exclusively data-driven studies, other authors reported using more traditional algorithms such as regression [27, 39], decision tree [78] or K Nearest Neighbors [75].

#### Evaluation and validation

The protocol to evaluate and validate the developed models was highly study-dependent and is detailed in the next subsections. The following characteristics were considered:

- The performance metrics;
- The comparison of results to other references;
- The use of external validation;
- The inclusion of a process to deal with the imbalance issues.

All these results are summarized in Table 4.

**Table 4:** Number of publications for different evaluation processes

Evaluation	Data driven	Knowledge-based	Hybrid
Comparison to other methods	15 studies	9 studies	4 studies
Comparison to other tools	1 study	8 studies	3 studies
Comparison to experts	3 studies	1 study	1 study
External validation	8 studies	8 studies	2 studies
Method for imbalance issue	2 studies	0 studies	0 studies
**Total**	**29 studies**	**19 studies**	**13 studies**

The number of studies is specified for each evaluation process according to the type of knowledge included. External validation is only specified in 18 studies, and a specific method to address imbalance issues is only specified in two studies

##### Metrics

In tasks associated with groups 2 and 3 (for differential diagnosis or diagnosis of all rare diseases), most of the authors calculated the proportion of correct diagnosis within the top K recommendations. K was chosen by the authors and varied greatly among the publications, from 1 (11 studies) to 100 (2 publications), with most articles using K = 10. Different values for K were generally assessed within each publication. Other studies, especially studies focusing on one disease (group 1) or a class of diseases (group 2), relied on various metrics, such as accuracy, F-score, positive predictive value (precision), sensitivity (recall) and specificity, number of correctly classified, false discovery rate or area under the receiver operating characteristic curve. These discrepancies in performance metrics make the comparison of results irrelevant.

##### Comparison to references

In 37 studies out of 61 studies (61%), the performance of developed diagnostic support tools was evaluated through a comparison to other references, which could be other methods (28 studies), preexisting tools (12 studies) or assessments from experts (5 studies). The other 24 studies did not report on a process of performance comparison of their developed tools.

##### External validation

Regarding validation:

- For data-driven systems, we assessed whether the algorithm was validated on an external dataset. Indeed, datasets can be subject to certain biases, and the methods can be overfitted to one dataset and fail in other datasets. Therefore, a validation step on an external dataset is required.
- For knowledge-based studies, we assessed whether models were evaluated on real patients.
- For hybrid models, both validation processes were considered.

Among 29 data-driven studies, only 8 studies described an external validation step. Four of these studies were published after 2017, whereas only one study was published before 2014, suggesting the increasing use of external validation sets. These external sets could consist of datasets from different centers [24, 33, 44, 53, 56, 57] or from the literature [43, 62].

Among 19 knowledge-based systems, only 8 studies validated their model on real cases [2, 46, 54, 55, 61, 67, 71, 77]. Among the 11 remaining studies, 5 studies considered simulated patients (queries consisted of a list of phenotype concepts). These studies aimed to diagnose genetic [63, 64, 68] and rare [66, 70] diseases from queries with phenotype concepts. Six studies did not provide any evaluation protocol [26, 32, 38, 50, 65, 69], among which four studies developed manually designed algorithms.

Among 13 studies using hybrid models, two used real datasets to test their models that were based on phenotype concepts for the diagnosis of rare diseases [73, 78]. The validation step of the other studies was limited to the original (internal) training and test sets.

##### Imbalance issue

Imbalance in sample size is a major issue that is common in the field of rare diseases. Two studies [47, 56] out of 61 proposed a method to address this issue. Interestingly, 2 antenatal studies out of 7 also proposed a method to address this issue. The proposed methods consisted of oversampling or downsampling. Some other studies considered adapting performance metrics to this imbalance issue, e.g., considering balanced accuracy [19, 33] instead of standard accuracy scores.

### Tool implementation

This section provides general information (such as intended users, tool maintenance) about the developed tools and algorithms and then a description of systems of interest considering three categories:

- Online tools
- Advanced tools/algorithms
- Innovative prototypes

#### General information

When specified, the diagnosis support systems were almost exclusively developed for clinicians. Except for Burange and Chatur [74], no system was intended to be used by the general public, even if some of them were freely accessible.

Maintenance/updating is an important issue for decision support systems. However, this process was clearly specified in very few cases. Updates were either based on manual review of new information by experts [54, 61] or automatically performed based on case reports retrieval from PubMed [71] or by downloading raw data from Orphanet [70].

We provide a description of the 14 ready-to-use tools, 18 advanced algorithms and 2 innovative prototypes.

#### Online tools

Studies led to the development of a tool with an identifiable name in 30 cases (29 different tools).

Fourteen systems were accessible (Table 5) using a provided URL, allowing us to test them or to download the code and data. All these tools or algorithms used phenotype concepts (12 tools) or images (2 tools). Except for IEMBase [54], which was dedicated to genetic disorders with inborn errors of metabolism, all tools based on phenotype concepts were generic tools for the diagnosis of all genetic or rare diseases. As discussed in the previous section, most of them considered providing patient recommendations based on the top K disease ranking. Despite the different values of K among studies, insights regarding their performances are given in Table 5 for the top 10 rankings. For each study, the performance score displayed in the table corresponds to the percentage of correct diagnoses encountered within the top 10 diseases suggested by the algorithm. When the exact value was not indicated by the authors but could be estimated through a figure provided within the study, an approximate value was given. These results should be interpreted with prudence as dataset nature and volume were quite dissimilar (e.g., datasets could consist of real or simulated patients).

**Table 5:** Characteristics of online tools

Tool name	Date	Data sources	Performances: Top 10 ranking	Related articles	URL
**Phenomizer**	2009	Phenotype concepts	NA	[63]	http://compbio.charite.de/phenomizer
**BOQA**	2012	Phenotype concepts	NA	[64]	http://compbio.charite.de/boqa/
**Phenotips**	2013	Phenotype concepts	NA	[65]	http://phenotips.org
**FindZebra**	2013	Phenotype concepts	63%	[66]	http://www.findzebra.com/
**PhenIX**	2014	Phenotype concepts/genes	~ 99%	[67]	http://compbio.charite.de/PhenIX/
**Phenolyzer**	2015	Phenotype concepts/genes	~ 85%	[69]	http://phenolyzer.usc.edu
**RDD**	2016, 2017	Phenotype concepts	38%	[2, 70]	http://diseasediscovery.udl.cat/
**IEMbase**	2018	Phenotype concepts	90%	[54]	http://www.iembase.org/app
**PubCaseFinder**	2018	Phenotype concepts	57%	[71]	https://pubcasefinder.dbcls.jp/
**RDAD**	2018	Phenotype concepts/genes	95%	[73]	http://www.unimd.org/RDAD/
**GDDP**	2019	Phenotype concepts	~ 32%	[77]	https://gddp.research.cchmc.org/
**Xrare**	2019	Phenotype concepts/genes	~ 95%	[78]	https://web.stanford.edu/~xm24/Xrare/
**CC-Cruiser**	2017	Images	NA	[44]	https://www.cc-cruiser.com/
**DeepGestalt**	2019	Images	NA	[62]	https://www.face2gene.com/

For each online tool, we listed the publication year, the materials used, the performance indicated in each publication, and the URLs provided in the publications. For the performance, the proportion of accurate diagnoses within the top 10 most relevant disease for each patient is given for all algorithms based on diagnoses recommendation (i.e., providing for each patient a list of potential diagnoses ranked by relevance). These results were provided by the authors of each tool and thus do not allow a comparison of tool performance, as the nature and volume of each dataset were different

Both systems using images used deep learning. One tool aimed at identifying congenital cataracts [44] from ocular images using a convolutional neural network, and the other study aimed at providing a facial image analysis framework to distinguish different genetic syndromes [62] from facial photographs.

Most of the tools using phenotype concepts relied on terminologies dedicated to rare diseases, including Orphanet vocabulary [79], OMIM terms [80] and HPO [81]. The Institute for Medical and Human Genetics from Berlin, which was involved in the development of the HPO [81], was also co-author of the following tools: Phenomizer [63], the BOQA (Bayesian Ontology Query Algorithm) [64], PhenIX [67] and Phenolyzer [69].

Eight models considering only phenotype concepts and four models considering both phenotype concepts and genotypes are described in the next sections.

##### Models including only phenotype concepts

The objective of **Phenomizer** [63] is to adapt semantic similarity metrics to measure phenotypic similarity between a patient represented by a set of phenotype concepts (query) and hereditary diseases described in a database and to develop a statistical model assigning *p* values to the resulting similarity scores. The p value is used to rank the candidate diseases. The ontology structure of HPO is used, and the similarity between a set of phenotype concepts and a disease is calculated based on the information content of their most informative common ancestor (MICA). The association between HPO terms and diseases from OMIM is considered. This method outperformed other scores in the simulated patient cohort.

**BOQA** [64] combines “ontological analysis with Bayesian networks to deal with noise, imprecision and attribute frequencies”. Queries are modeled through a three-layered Bayesian network of Boolean variables. HPO frequencies are included in the model. The performance was also tested on simulated patients.

**Phenotips** [65] proposes a deep phenotyping tool that suggests a ranked list of disorders using similarity measures on phenotype concepts encoded with HPO. It accounts for negative phenotype concepts and disorder frequency (extracted from Orphanet). The article doesn’t mention any evaluation.

**FindZebra** [66] is a search engine dedicated to rare diseases that uses a query corresponding to a combination of phenotype concepts to propose a ranked list of documents from specialized resources. Documents are ranked using a state-of-the-art query likelihood ranking model. The document is considered relevant if it predominantly addresses the correct disease. The system was compared to generic search systems such as Google or PubMed and outperformed them on a test set consisting of 56 queries created by clinicians or based on clinical cases from published articles.

**Rare Disease Discovery** (RDD) [2, 70] aims to aid in the initial diagnosis of rare diseases using a user-friendly web application. The system integrates a mapping between Orphanet and HPO terms and a scoring function for disease ranking based on the number of phenotype concepts in common between the query and the tested disease. The authors tested different parameters (e.g., minimum statistically significant value for the score) and compared their prototype to other systems in terms of the top 10 rankings of correct disease [2] and to other methods (machine learning, Bayesian networks) and other tools [70].

**PubCaseFinder** [71] aims to increase the coverage of DPA (disease-phenotype associations) databases and consequently improve the performance of differential diagnosis systems for rare diseases. From a list of queried phenotype concepts, the system provides a disease ranking based on DPA extracted from PubMed and from Orphanet using a similarity measure based on Information Content (GeneYenta). The system was compared to existing tools.

**GDDP** (Genetic Disease Diagnosis based on Phenotypes) [77] aims to improve the accuracy of matching rare genetic diseases based on patient phenotype concepts. Prioritization is either based on similarity metrics using MICA and considering a null similarity for terms not on the same lineage or using ontological overlap. Performance was evaluated on both simulated patients and medical records and compared with existing tools.

**IEMbase** [54] is a prototype mini-expert system for diagnosis support, combining the inborn errors of the metabolism community knowledge base. The specificity of the study is that the model differentiates between “clinical phenotypes” and “biochemical phenotypes”. Different structured vocabularies (e.g., HPO, Logical Observation Identifiers Names and Codes, Systematized Nomenclature of Medicine–Clinical Terms) for matching with these two categories of phenotype concepts were tested. An algorithm was developed using weighted cosine similarity for biochemical phenotypes and semantic similarity for clinical phenotypes. The best results were obtained with the combination of both types of phenotype concepts. The tests were performed using retrospective cases.

##### Models including phenotype concepts and genes

**PhenIX** [67] combines queries using phenotype concepts to genetic information for the diagnosis of Mendelian diseases. For each patient, variants are identified through the sequencing of the Disease-Associated Genome (DAG). The system suggests associated diseases/genes by ranking variants based on pathogenicity and semantic similarity of patients’ phenotype concepts. Evaluation was carried out on simulated and real patients.

**Phenolyzer** [69] integrates information from phenotype, disease and gene databases to prioritize human disease genes based on disease or phenotype information provided by users as free text. For disease matching, HPO frequencies and conditional probabilities from OMIM are considered. The system first identifies the associated disease and then prioritizes genes using a disease-gene score including different parameters. Disease prioritization was compared to Phenomizer for 14 monogenic diseases and led to comparable results.

**RDAD** (Rare Disease Auxiliary Diagnosis system) [73] aimed to build diagnostic models using phenotypic similarity and machine learning. Models using information from different databases (phenotypes-disease, phenotype-gene, text-mined disease-phenotype associations) and different similarity methods (including or excluding machine learning) were compared. Real medical records were used for evaluation. All the methods are available and can be tested using the RDAD web application.

The **Xrare** tool [78] aims to prioritize causative gene variants in rare Mendelian disease diagnosis. The model includes information from variant databases, guidelines for variant prioritization, and gene-phenotype associations. The model uses machine learning from 51 features derived from these data to predict the causative variant and the associated disease. These features include similarity scores between sets of phenotype concepts. The system was tested on simulated data and real clinical data sets. The proposed emission-reception information content score ranked consistently higher for disease genes than other phenotypic similarity scores in the presence of imprecise and noisy phenotype concepts.

#### Advanced tools and algorithms

Eighteen studies led to the implementation of a model intended to be ready to use in clinical routine, including 7 manual tools. These manual tools consisted of scores, decision trees and guidelines for the diagnosis of Marfan syndrome [25, 27], Fabry disease [26], diseases with recurrent wheals or angioedema [50], HNF1B-related disease [32], familial chylomicronemia syndrome [38] and Niemann-Pick disease Type C [39]. The 11 remaining tools are described in the following subsections.

##### Routinely usable tools

Six tools combining multiple or new techniques and machine learning methods were intended to be (according to their authors) less time consuming, less costly, or more accurate alternatives to current diagnosis processes. Among them, four combined spectroscopy based on disease-related fluid data (urine or blood samples) with machine learning. Three of them were published between 2009 and 2013, taking advantage of the development of proteomics and aimed to support the diagnosis of cystic fibrosis [48], thalassemia [20] and hereditary hemorrhagic telangiectasia [31]. Another method was proposed in 2019 [60] for the differential diagnosis of mucopolysaccharidoses and subtype classification. Two other studies [28, 29] proposed systems for less expensive diagnosis of thalassemia combining real-world data obtained in routine analysis and artificial neural networks.

##### Differential diagnosis

Distinguishing between complex rare diseases with overlapping phenotypes can be challenging. This issue was addressed by three tools for the identification of 6 rare pulmonary diseases with common chronic cough [52] using a questionnaire, congenital upper-limb abnormalities [55] based on hand phenotype concepts and genetic syndromes [43] based on facial photographs. Comparison to human experts led to comparable performances [41, 43].

##### Improving preprocessing

Data heterogeneity can bring biases to the analysis and have an impact on tool performance and reproducibility. Thus, some works have focused on improving preprocessing to improve classification performances. Kostro et al. [33] worked on improving the early detection of neurodegenerative brain diseases based on scanner images by correcting the effects of subject-specific covariates (such as age, total intracranial volume, and sex) as well as inter-scanner variability by using a nonlinear Gaussian process. The process was tested for the classification of carriers of the genetic mutation leading to Huntington’s disease. Natarajan et al. [58] worked on the issue of recruiting patients for a clinical study based on active feature elicitation. Four real clinical tasks were considered, including the prediction of rare diseases from a survey.

#### Innovative prototypes

Twenty-nine studies described prototypes that needed further validation or improvement to be considered mature. Some of these prototypes proposed novel approaches that could lead to interesting tools in the future. For example, the ADA DX prototype [61] includes the temporality for symptom discovery to assess the possibility of accelerating the diagnosis. For each patient, the system proposes a diagnosis per visit, using only evidence from the associated documents. The time to accurate diagnosis for the system is then compared to the time to diagnosis in real life. Another interesting system was the only fully data-driven method based on phenotype concepts identified in our review [72, 75]. This system consists of two versions that were described in two different studies. In the first version [72], no expert knowledge was included, and data were extracted from the EHR, whereas in the second version [75], EHR data were combined with knowledge extracted from medical literature. Machine learning algorithms were applied to cluster patients based on different similarity measures. In this case, contrary to most studies, similarities were measured between patients and not between a patient and a disease.

## Discussion

### Overview

The development of diagnosis support tools for rare diseases has gained more interest in recent years (2 articles were published in 2009 compared with 12 articles in 2018). One recent review published in 2019 [83] aimed to analyze AI solutions in rare diseases but did not specifically focus on diagnosis support systems. Moreover, they did not include all methods based on similarity measurements between patients and rare diseases.

In this scoping review, we restricted the time period to the last ten years, and only publications in English were considered. Both PubMed and WoS were used for identifying relevant studies, and three AI conference websites were explored as well. The search queries were tailored for each source.

We completed the selection by screening the bibliographies of relevant publications and “similar articles” suggested by PubMed. The unpublished tools (developed by industrial companies only for commercial use) were not included.

Publications were clustered into three groups according to their objective with respect to the number of targeted diseases. Approximately one-third of studies aimed at providing a diagnosis to all rare or genetic diseases. Most of these generic tools relied on phenotype concepts and aimed at providing expert knowledge to nonexpert clinicians to tackle misdiagnosis and delay in diagnosis. In a few cases, these phenotype concepts were combined with genetic data. Studies focusing on classes of diseases or unique diseases could take advantage of disease-related materials (such as fluids or images).

Regarding the methods, expert knowledge was included in half of the studies (hybrid or knowledge-based approaches). Machine learning was really widespread and was used in approximately two-thirds of the systems. Neural networks and SVM were the most common machine learning methods. Neural networks were mostly used with fluids or images in studies including at least several hundred patients. Other considered methodologies consisted of simple similarities, generally applied to phenotype concepts, and manual algorithms.

Regarding implementation, three categories of algorithms and tools were found according to their maturity and level of accessibility: online tools, advanced tools or algorithms and prototypes. Online tools were mostly diagnosis tools for all rare or genetic diseases working with phenotype concept queries. In addition to providing differential diagnosis to important groups of diseases, the advanced tools also included proposals for more routinely usable tools (aiming at providing less expensive, less time consuming, easier to use or insightful solutions) or for better preprocessing processes. Prototypes were not fully described, but a presentation of two recent innovative prototypes with nontraditional approaches was provided.

### Technical significance

Numerous tools are freely accessible online and can be tested and used by researchers and clinicians. However, accessibility raises the question of update and maintenance, which was sometimes ignored or not described in the article. Indeed, among the 18 online tools, 4 were not accessible using the URL provided in the publication and could not be found via standard search engines, and one tool URL had been modified. Moreover, as previously mentioned, how the tools were updated was barely specified, whereas for example, terminologies such as HPO evolve considerably over time, e.g., including new terms.

Regarding tool maturity, 32 studies (52%) led to systems that were considered ready to use. The remaining 29 initiatives corresponded to tools that generally needed further validation or improvement to reach better performances. Regarding the latter, it is not clear whether development is still ongoing, as approximately two-thirds of the studies describing these prototypes were published before 2018, and no new publication has been found regarding these tools since then. Moreover, some tools that were considered by their authors as ready to use and accessible had only been tested on simulated patients or calibrated on test sets, which is a limitation in considering these tools as completely mature.

For data-driven systems, the lack of explicability of models brings even more uncertainty and makes it more difficult to identify biases due to dataset constitution, whereas more “explainable artificial intelligence” is sought by regulators as a guarantee of trust and transparency. Combining expert and data knowledge could be a good way to enhance the explicability of developed models.

### Clinical significance

Most studies based on phenotype concepts considered HPO for encoding. The fact that a common ontology has been adopted by all researchers is a really positive aspect that facilitates the possibilities for interoperability and tool comparison. One possible limitation is still the language adaptation, since in non-English speaking countries, a system that would be interfaced with a medical database needs detecting and encoding phenotype concepts in this language. However, the HPO terminology is not as developed in other languages as it is in English. This terminology would need to be fully extended to other languages so that these tools can be fully used by everyone.

The reported performances were generally good. Regarding online tools, in 5 studies out of 9, the correct diagnosis was found within the top 10 suggestions in more than 85% of the cases. Similarly, the accuracy of the two online tools based on images [44, 62] was higher than 89%. However, more generally, the evaluation metrics were far from standardized, making the comparisons difficult. For example, when the proportion of accurate diagnosis within the top K recommendations was used, K was not the same in all publications, ranging from 1 to 100. Such heterogeneity is an obstacle to easy interpretation and comparison of results. Other studies relied on numerous and various metrics, such as accuracy, F-score, precision and recall.

Moreover, as previously stated, only a few studies validated their findings on external datasets. This raises major concerns, as variability of data quality is important when considering real-life evidence. A mismatch between the datasets used for developing the algorithm and the characteristics of the population targeted by the system can inadvertently introduce biases, most commonly by deficiencies in the training data but also by application of the system to an unanticipated patient context [84]. In addition, training sets used for machine learning models were generally not publicly available. Regarding knowledge-based models, the majority of developed models and algorithms (generally manual models) were not evaluated on real patients. Moreover, some studies [67, 77] obtained performances that were far less good on real patients than on simulated ones. For example, when tested on simulated patients with different levels of noisy phenotype concepts, Phenomizer classified at least 75% of correct diseases as top 1, whereas when tested by another team [77], the method used in Phenomizer (best match average method) on a dataset consisting of 462 EHRs reached less than 10% of the top 10 correct disease rankings. The impact of changing the dataset for evaluation was confirmed when Phenomizer was tested for comparison with developed tools in numerous studies and obtained results that highly depended on the dataset under study [2, 55, 68–71, 77].

One positive aspect is that the availability of these tools enables comparison on new datasets by potential future users. Before choosing one of these tools, we recommend comparing and validating them on external datasets.

Of note, the only system that integrates data from the EHR in an automated manner is the future MIRACUM project [76].

### Perspectives

Numerous initiatives benefiting from AI are enabled by the progressive coverage of EHR systems and are developing with interesting results. Comparison with human experts sometimes led to comparable results, which underlines the perspectives of such initiatives. However, an important limit to the development of AI solutions in the field of rare disease diagnosis is the data volume. Indeed, 9 studies out of 10 using neural networks benefited from several hundred rare disease records, which may be seen as an important volume in this field. More generally, validation on small datasets, especially with imbalance issues, raises the question of performance significance. The methods need to be adapted to take into account these limitations, e.g., taking advantage of new methods such as transfer learning, which is particularly adapted when the volume of the training set is limited.

In the coming years, AI has the potential to facilitate early detection of rare diseases, especially for patients who could not have easy access to experts. Most tools were intended for clinicians, meaning that the objective of these tools is to be used in clinical routine. To reach this goal, these tools need to be adequately evaluated. Moreover, tools intended to be plugged into EHR systems need to be interoperable and adapted to EHR analysis. For example, some variables used in the diagnosis support system may be present in the EHR in unstructured format, e.g., in narrative reports.

Suggestions to allow more widespread use of proposed methodologies and accurate models include:

- To use standardized metrics to facilitate evaluation and comparison. For studies using the top K ranking of possible diagnoses, we recommend providing at least the top 10 disease rankings and the mean ranking of correct disease over all patients.
- To use standardized terminologies to enhance interoperability and spread of the tools. For systems based on phenotype concepts, we recommend using the HPO, provided that this terminology keeps being enriched and is available in several languages.
- To combine expert and data knowledge to enhance explicability
- To provide robust methods dealing with the imbalance and data volume issues
- To make training sets accessible
- To validate the findings on external datasets and real patient cases
- To measure the impact on patient diagnosis and outcomes.

## Conclusions

Clinical diagnosis excellence in the field of rare diseases demonstrates the societal need and opportunity to develop AI technologies. This scoping review was conducted to identify algorithms and tools to support the diagnosis of rare diseases. This overview enabled the identification of various approaches relying on various materials and methods. Numerous solutions are emerging with satisfying preliminary results. However, the variability of approaches and evaluation processes complicates the comparison of results. Efforts should be made to adequately validate these tools and guarantee reliability, reproducibility, explicability and interoperability so that these tools can be safely used in clinical settings.

## Data Availability

Data sharing is not applicable to this article, as no datasets were generated or analyzed during the current study. All articles reviewed for this study are mentioned in this published article.

## Abbreviations

AAAI: Association for the Advancement of Artificial Intelligence
AI: Artificial Intelligence
AIJ: Artificial Intelligence Journal
BOQA: Bayesian Ontology Query Algorithm
DPA: Disease-Phenotype Associations
EHR: Electronic Heath Record
GDDP: Genetic Disease Diagnosis based on Phenotypes
HPO: Human Phenotype Ontology
IJCAI: International Joint Conferences on Artificial Intelligence
JAIR: Journal of Artificial Intelligence Research
MeSH: Medical Subject Heading
MICA: Most Informative Common Ancestor
ML: Machine Learning
NeurIPS: Neural Information Processing Systems
OMIM: Online Mendelian Inheritance in Man
RDAD: Rare Disease Auxiliary Diagnosis system
RDD: Rare Disease Discovery
SVM: Support Vector Machine
WoS: Web of Science
